# Buffering or Backfiring? A Meta-Analysis of the Effects of Corporate Social (Ir)responsibility on Firm Risk

**DOI:** 10.1177/00076503251350044

**Published:** 2025-06-29

**Authors:** Won-Yong Oh, Rong (Ratchel) Zeng, Maoliang Bu

**Affiliations:** 1 University of Nevada, Las Vegas, USA; 2 University of Manitoba, Winnipeg, Canada; 3 Nanjing University, China

**Keywords:** corporate social irresponsibility, corporate social responsibility, firm risk, meta-analysis

## Abstract

Corporate social responsibility (CSR) and irresponsibility (CSIR) impact firm risk in complex, dynamic ways, rather than as mere opposites. In a meta-analysis of 197 empirical studies, we find asymmetric patterns in which CSIR’s risk-generation effects outweigh CSR’s risk-mitigation effects. These impacts vary across dimensions of social outcomes, risk types, and contextual moderators. Moreover, CSR’s risk-mitigation effects are influenced by indirect paths related to CSIR, whereas CSIR’s risk-generation effects are not associated with indirect paths. Our findings highlight the nuanced interplay among CSR, CSIR, and risk, offering new insights for future research on theoretical mechanisms, contexts, measurements, and methods.

Corporate social responsibility (CSR) and corporate social irresponsibility (CSIR) have emerged as pivotal constructs in the research on corporate social performance (CSP). The growing attention from both scholars and practitioners reflects a broader shift in how firms navigate social and ethical imperatives while managing financial and operational risks (Clark et al., 2022; R. Fu et al., 2020). Over the years, empirical studies have explored the implications of CSP across multiple domains, prompting meta-analyses to synthesize these findings and clarify its effects on firm-level outcomes (Wang et al., 2016). Among these organizational outcomes, firm risk stands out as a particularly critical measure, capturing the likelihood of financial instability, market volatility, and potential failure (Godfrey et al., 2009; Orlitzky & Benjamin, 2001). Understanding the link between CSP and firm risk is both significant for theoretical reasons and urgent for practical ones, given the growing complexity of corporate decision-making.

Although there is a growing body of research on CSP and firm risk, the literature remains fragmented. Prior studies provided insights into CSP-risk relationships under specific contexts. These studies (the “trees”) are limited to context-dependent characteristics, which prevents a comprehensive understanding of the entire picture (the “forest”). Most studies have examined the CSR-risk relationship and CSIR-relationship separately, limiting our understanding of their relative effects and underlying dynamics. Meanwhile, numerous studies have treated CSR and CSIR as opposite ends of the same spectrum (e.g., calculating CSP scores by subtracting CSIR from CSR), which risks misrepresenting these two distinct constructs. As a result, it has been challenging to comparatively evaluate CSR’s risk-mitigation and CSIR’s risk-generation effects (i.e., *which effect is greater?*). Moreover, existing research often employs a bivariate approach, analyzing the CSP-risk linkage in isolation, even while acknowledging that CSR and CSIR are conceptually distinct, yet clearly interrelated. This oversimplification overlooks critical indirect pathways—such as the interplay between CSR and CSIR—that shape firm risk, thereby leading to either an overestimation or underestimation of the effects of CSR and CSIR on risk. While there have been studies examining the various impacts of CSR and CSIR on firm risk in different contexts, few have systematically synthesized these findings to provide clear directions for future research.

Our study addresses the gaps described above and contributes to the literature in several ways. First, by conducting a meta-analysis of 197 empirical studies, we provide a systematic synthesis that offers a “forest-level” view of CSP-risk dynamics, moving beyond fragmented context-specific findings. Furthermore, these effects vary across different CSP dimensions (i.e., environmental, social, and governance activities), risk types, and several moderators, suggesting that a one-size-fits-all approach to CSP and risk management is insufficient. Our study advances the understanding of CSR and CSIR by moving beyond context-specific findings and toward a more comprehensive perspective, highlighting the differential impacts of CSP activities on firm risk. Second, our findings reveal an asymmetric pattern in which CSIR’s risk-generating effects outweigh CSR’s risk-mitigation effects. It directly compares CSR’s risk-mitigation effects and CSIR’s risk-generating effects, revealing the stronger impact of the latter. Third, using meta-analytic structural equation modeling (MASEM), we find that CSR’s risk-mitigating effects on firm risk are partially mediated by CSIR (i.e., the suppressor effect), supporting an indirect pathway. In contrast, CSIR’s risk-generating impact shows no evidence of indirect paths through CSR. This finding challenges traditional *bivariate* approaches by adopting a *multivariate* lens, recognizing the complex interdependencies between CSR, CSIR, and risk outcomes. Lastly, drawing on the findings from our meta-analysis, our study identifies key directions for future inquiry, offering theoretical and methodological guidance for advancing CSP-risk scholarship.

## Theory and Hypotheses

### CSR and CSIR: Conceptual Distinctions

Attention to CSR and CSIR as key elements of CSP^1^ has grown in both theory and practice (Fu et al., 2020; Li & Wu, 2020; Qian et al., 2019). CSR refers to “actions that appear to further some social good, beyond the interests of the firm and that which is required by law” (McWilliams & Siegel, 2001, p. 117), involving proactive efforts to meet economic, social, and environmental objectives. In contrast, CSIR is defined as a “set of corporate actions that negatively affects an identifiable social stakeholder’s legitimate claims” (Strike et al., 2006, p. 852) or fail to meet societal expectations (Clark et al., 2022).

Although firms often engage in both CSR and CSIR (Strike et al., 2006), they are conceptually distinct and should be examined separately (Clark et al., 2022). Treating them as opposite ends of the same continuum, such as by calculating CSP scores by subtracting CSIR from CSR empirically, risks conflating two distinct constructs, thereby obscuring the distinct effects of CSR and CSIR on firm risk. To address this methodological issue, we examine CSR and CSIR as conceptually related but independent constructs, allowing for a more accurate estimation of their respective impacts on firm risk (Crane et al., 2018).

### CSR’s Risk Mitigation and CSIR’s Risk Generation

#### Risk-Mitigation Effects of CSR

CSR acts as a risk-management tool by addressing stakeholder concerns, preventing conflicts, and building support (McWilliams & Siegel, 2001). It reduces risks through both preventative (ex ante) and protective (ex post) benefits. Proactive CSR activities, such as sustainable practices and improved governance, lower the likelihood of negative events (ex ante) like pollution or scandals by building stakeholder trust, creating a “prevention effect” (Jia et al., 2020). Additionally, CSR provides insurance-like protection against adverse events by generating moral capital, such as investor trust and customer loyalty, to soften the impact of crises (ex post) (Godfrey, 2005). Peloza (2006) likened CSR to purchasing insurance for a firm’s reputation, mitigating the harm of negative publicity. Although some argue that firms that are perceived as socially responsible may face heightened stakeholder expectations and thus greater risk (Fu et al., 2022), the prevailing view holds that CSR is negatively associated with firm risk (Godfrey, 2005). This view is supported by empirical evidence showing that CSR helps reduce market, credit, and financial risks (Godfrey et al., 2009; Shiu & Yang, 2017).

**Hypothesis 1:** There is a negative relationship between a firm’s CSR and risks.

#### Risk-Generation Effects of CSIR

CSIR is a key driver of firm risk (Kölbel et al., 2017) as it undermines a firm’s legitimacy and reputation (Windsor, 2013). Irresponsible business decisions fail to secure stakeholder support and often provoke retaliatory actions, such as negative publicity and consumer boycotts (Distelhorst & McGahan, 2021). Stakeholders tend to react strongly to negative incidents, driven by a desire to punish the firm and deter future irresponsibility (Kölbel et al., 2017), creating financial, credit, and reputational risks. Additionally, firms with high levels of CSIR are often less equipped to mitigate risks, making them more vulnerable to severe stakeholder sanctions (Godfrey, 2005). Although stakeholder responses vary and CSIR incidents may fade over time (Barnett, 2014; L. Fu et al., 2022), we argue that the risk-generating role of CSIR remains salient due to its enduring impact on stakeholder trust and firm vulnerability.

**Hypothesis 2:** There is a positive relationship between a firm’s CSIR and risk.

### Positive Relationship Between CSR and CSIR

We argue that CSR mitigates risk while CSIR generates it; however, when considering the covarying effects of CSR and CSIR, their implications for firm risk differ and are more complex. Firms often engage in both simultaneously (Strike et al., 2006), implying a positive relationship between the two (i.e., doing both good and bad). For instance, firms may use CSR to offset CSIR, a strategy referred to as the “penance mechanism” (Kang et al., 2016). In contrast, CSR may also foster CSIR through “moral licensing,” enabling less ethical behaviors (Ormiston & Wong, 2013). These lines of research view the causal link between CSR and CSIR differently but agree that the two constructs positively covary (Nguyen et al., 2024).

**Hypothesis 3:** There is a positive relationship between a firm’s CSR and CSIR.

### Countervailing Mechanisms

CSR and CSIR are interrelated, with studies suggesting their countervailing effects (Clark et al., 2022), though the mechanisms behind these dynamics remain unclear (Murphy & Schlegelmilch, 2013). We propose that CSR’s risk-mitigation effects vary based on “indirect” paths, namely, (a) its linkage with CSIR and (b) CSIR’s risk-generation effects. Similarly, CSIR’s risk-generation effects depend on (a) its linkage with CSR and (b) CSR’s risk-mitigation effects. These dynamics are crucial as firms often engage in both behaviors (Strike et al., 2006). We hypothesize that CSR’s direct risk-mitigation and CSIR’s direct risk-generation effects are weakened by the underlying countervailing mechanisms between CSR and CSIR (i.e., the suppressor effect).

#### Diminished Risk Mitigation Hypothesis

We propose indirect paths linking CSR to firm risk through CSIR. Firms with high CSR often exhibit high CSIR (Hypothesis 3), often leading to stakeholder skepticism (Ogunfowora et al., 2018). Empirical evidence shows that firms engaging in CSR frequently practice CSIR (Strike et al., 2006), as CSR may provide a sense of moral licensing, justifying less responsible behaviors (Ormiston & Wong, 2013). This implies that although CSR directly reduces firm risk, its positive association with CSIR may indirectly increase risk, thereby offsetting some of the benefits of CSR. As an increase in CSIR generates risk (Hypothesis 2), we argue that CSR’s risk-mitigation effects weaken due to these countervailing mechanisms. Accordingly, the overall impact of CSR on firm risk is shaped not only by its direct effects but also by its indirect effects via CSIR, which may undermine its protective value.

**Hypothesis 4:** CSR’s direct effects of risk mitigation weaken when considering the indirect effects (i.e., outcome of CSR-CSIR linkage and CSIR-risk linkage).

#### Diminished Risk Generation Hypothesis

To address CSIR’s risk-generating effects, firms often use CSR to offset prior CSIR, known as the “penance mechanism” (Kang et al., 2016). This strategy mitigates risk by counterbalancing CSIR activities, though it may face criticism as being a form of greenwashing and redirect stakeholder focus away from CSIR. We propose that there are indirect paths linking CSIR to firm risk via CSR, as a countervailing mechanism that offsets the risk-enhancing effects of CSIR. The penance mechanism implies a positive CSIR-CSR relationship (Hypothesis 3), and since CSR mitigates risk (Hypothesis 1), CSIR’s risk-generation effects are offset by CSR’s risk-mitigation effects. Given the positive relationship between CSIR and CSR, and the established risk-mitigating role of CSR, we argue that this indirect path helps to offset some of the adverse impacts of CSIR.

**Hypothesis 5:** CSIR’s direct effects of risk generation weaken when considering the indirect effects (i.e., outcome of the CSIR-CSR linkage and CSR-risk linkage).

### Decomposing CSP and Risk Constructs

When managing CSP, firms should determine which areas to prioritize and how to address varying risks. Rather than treating CSP and firm risk as singular constructs, we decompose them into dimensions, as both are multi-dimensional constructs.

#### Environmental, Social, and Governance Dimensions of CSR and CSIR

Prior research (Clark et al., 2022) suggests that breaking CSR and CSIR into specific dimensions provides insight into a firm’s social motives, processes, and outcomes. Building on prior studies (Kölbel et al., 2017; Li & Wu, 2020), we focus on environmental, social, and governance (ESG) dimensions. These dimensions influence stakeholders differently, affecting firm risk in unique ways (Bouslah et al., 2013), and therefore, it is important to analyze how each ESG dimension uniquely affects firm risk.

#### Different Types of Firm Risk

Similarly, our meta-analysis examines various types of firm risks, including market, financial, and operational risks (Lipsey & Wilson, 2001). Market risk involves losses in firm value and is often reflected in stock price volatility (Kölbel et al., 2017). Financial risk pertains to a firm’s inability to repay debt, typically measured by its debt ratio (Zyglidopoulos et al., 2012). Operational risk refers to losses from flawed processes or systems, often measured by cash flow volatility and operational leverage (Fiaschi et al., 2016).

### Exploratory Moderator Analyses

**Figure 1.**
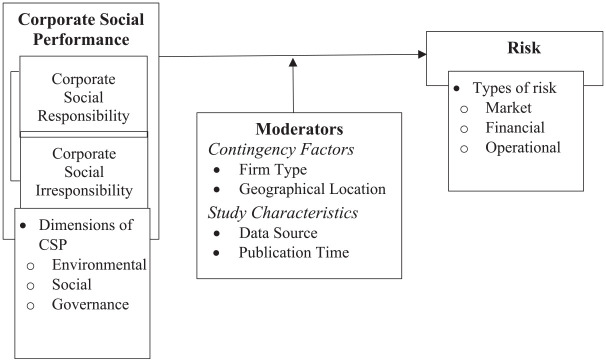
Conceptual Framework.

The exploratory approach in meta-analysis offers several advantages, such as examining inconsistencies in prior findings, correcting sampling and measurement errors, and in particular, detecting the characteristics of original studies as potential moderators (Kirca et al., 2012). Our moderators can be classified into two groups: *contingency factors* and *study characteristics*. The first covers sample attributes (e.g., location, firm type), while the second focuses on methodological aspects (e.g., data sources, publication time), aligning with prior meta-analyses (Geyskens et al., 2006). In Figure 1, we present the conceptual framework.

## Method

### Sample and Data

We followed the PRISMA guidelines (Moher et al., 2009) to identify studies focusing on both CSR and CSIR and their relationship with risk for our meta-analysis. Our study distinguishes itself from previous related studies (Orlitzky & Benjamin, 2001; Wang et al., 2016) by ensuring that the papers we included align with the current literature’s recommended operationalization of CSR and CSIR (Clark et al., 2022; Lin-Hi & Blumberg, 2018). As a result, studies that operationalized CSR as the difference between CSR and CSIR (e.g., strengths minus weaknesses) were excluded (Cai et al., 2012). We included only studies that reported correlations either between CSR and CSIR or between CSR/CSIR and risk.

To achieve this, we followed a multistep process that included several stages: search, preliminary screening, coding and further screening, and analysis. Each step is described in detail in Supplemental Appendix A. Ultimately, we included 181 papers with 197 studies in our final meta-analysis dataset (some papers included multiple empirical studies), covering 222,845 firms and 1,285,635 observations. The full list of papers included in the meta-analysis is provided in Supplemental Appendix E. Our sample is the most comprehensive to date, exceeding 10 times the number included in previous meta-analytic reviews of CSP and risk (Orlitzky & Benjamin, 2001).

### Meta-Analytic Procedures

The main variables, CSR and CSIR, were treated as multidimensional constructs, allowing for decomposition and subgroup analysis. Firm risk was subdivided into market risk, financial risk, and operational risk. While some studies focus on a single CSR/CSIR dimension or a specific type of risk, others examine two or more dimensions. Following standard meta-analysis practices, we aggregated these variables into a composite index for the main effect analysis using the Schmidt and Hunter (2015) method. However, we also coded them separately to enable meaningful comparisons in the subgroup moderator analysis (Steel et al., 2008). The key variables and their descriptions are summarized in Table 1. A key characteristic of our analysis is ensuring that CSR and CSIR operationalizations align with the current literature. Therefore, we carefully examined how these constructs were measured to ensure that the meta-analyzed studies did not treat CSR and CSIR as opposite constructs (see Supplemental Appendix B for details and Table B1 for examples of each measurement).

**Table 1.** Key Constructs and Examples of Measures.

Constructs	Examples of measures
CSR	Index as the sum of all scores for indicators identified as CSR, CSP strength, or CSR strength; weighted average of sub-scores for CSP or CSR strengths across different dimensions; positive activities that may help firms achieve CSR goals.
Environmental CSR	CSP or CSR strengths for environmental dimensions; environmental investments; weighted average of sub-scores for CSP or CSR strengths across environmental dimensions.
Social CSR	CSP or CSR strengths for social dimensions; weighted average of sub-scores for CSP or CSR strengths across social dimensions; investments in community, diversity, employees, or social policies.
Governance CSR	CSP or CSR strengths for governance dimensions; weighted average of sub-scores for CSP or CSR strengths across governance dimensions.
CSIR	Accumulated number of CSIR events, wrongdoing, misconduct, or controversies in which a firm has been involved; index as the sum of scores for CSP weaknesses or CSP concerns; engagement in irresponsible activities such as corruption.
Environmental CSIR	Concerns or weaknesses in environmental issues, pollution; hazardous waste, total emissions, and environmental misconduct.
Social CSIR	Concerns or weaknesses in social issues, such as community, diversity, and employees; human rights abuse controversies; or events.
Governance CSIR	Concerns or weaknesses in governance issues; tax avoidance proxies/measures; enforcement actions of securities regulatory commission.
Risk	All types of risk, such as market risk, financial risk, or operational risk, or composites of different types of risk.
Market risk	Idiosyncratic risk, as reflected in fluctuations of stock prices, credit default swaps (traded derivatives); variation/volatility of daily stock price; modeled stock responses.
Financial risk	Risk from the inability to pay debt, as reflected in the ratio of total liabilities to total tangible assets; debt to asset ratio: debt to equity.
Operational risk	Risk from fluctuations of future expected cash flows, operations, and volatility of return on equity, cash volatility, operational leverage.
Sample characteristics	
Data source	Database = 1, if the study uses archival data from databases such as KLD, ASSET 4, Sustainalytics, etc. Database = 0, if the study uses non-archival data such as interviews and questionnaires.
Publication time	Publication year = 1, if the publication year is before 2010. Publication year = 0, if the publication year is during or after 2010.
Firm type	Non-MNE = 0, if all the sampled firms are domestic firms. MNE = 1, if all the sampled firms are MNEs.
Geographical location	US = 1, if the sampled firms are located in the United States. US = 0, if the sampled firms are not located in the United States.

*Note*. CSIR = corporate social irresponsibility; CSP = corporate social performance; CSR = corporate social responsibility; MNE = multinational enterprise.

We followed rigorous meta-analysis methods (Geyskens et al., 2009) and established approaches (Schmidt & Hunter, 2015) to correct for measurement and sampling errors. For each pairwise relationship, we used random-effects estimation to calculate coefficients, standard errors, confidence intervals, credibility intervals, and other statistics. We employed weighted least squares regression for the moderator analysis due to its robustness in addressing multicollinearity and heteroscedasticity (Steel et al., 2008). Additionally, we conducted MASEM to explore and synthesize the relative impact of CSR and CSIR on risk. Further details on the MASEM procedures are provided in Supplemental Appendix B.

## Results

Table 2 presents the main meta-analytic findings on the correlations among CSR, CSIR, and risk, along with the subgroup analyses for moderators. It shows the amount of variance explained by moderators and the statistical significance of subgroup differences (*R*^2^ and *F* columns).

**Table 2.** Meta-Analytic Results for CSR, CSIR, and Risk.

Variable	*K*	*N*	*r*	*SD_r_*	*Q*	*R* ^2^	*F*	90% Cr. I_1_
CSR: Risk	101	136,360	−0.008*	0.069	591.88			[−0.11, 0.10]
Type of CSR						0.135	18.33***	
Environmental	11	6,735	0.044***	0.056	21.27			[−0.02, 0.11]
Social	11	13,934	0.055**	0.096	134.03			[−0.10, 0.21]
Governance	4	1,509	−0.067**	0.096	6.97			[−0.14, 0.01]
Type of risk						0.150	7.88***	
Market	16	10,245	−0.118***	0.089	81.79			[−0.25, 0.01]
Financial	96	111,121	0.011**	0.069	535.00			[−0.09, 0.11]
Operational	7	3,415	−0.021*	0.036	4.37			[−0.02, 0.02]
CSiR: Risk	134	167,067	0.063***	0.078	1,031.71			[−0.06, 0.18]
Type of CSIR						0.089	2.48*	
Environmental	14	11,493	0.078***	0.089	94.97			[−0.06, 0.21]
Social	14	16,025	0.057***	0.063	63.20			[−0.03, 0.15]
Governance	15	22,736	0.051***	0.048	52.22			[−0.01, 0.12]
Type of risk						0.044	3.35***	
Market	21	15,693	−0.015**	0.071	63.59			[−0.11, 0.08]
Financial	132	168,198	0.063***	0.079	1,115.56			[−0.06, 0.19]
Operational	8	6,036	0.067*	0.098	59.23			[−0.08, 0.22]
CSR: CSIR	145	144,274	0.258***	0.156	4,183.33			[0.01, 0.51]
Type of CSR/CSIR						0.246	11.46***	
Environmental	17	8,694	0.322***	0.090	85.00			[0.19, 0.45]
Social	14	12,207	0.130***	0.102	135.15			[−0.03, 0.29]
Governance	6	2,965	0.055	0.275	259.66			[−0.39, 0.50]

*Note*. Cr. I = credibility interval; CSIR = corporate social irresponsibility; CSP = corporate social performance; CSR = corporate social responsibility; *k* = number of studies; MNE = multinational enterprise; *N* = sample size in number of firms; *r* = weighted and reliability corrected correlation; *SD_r_* = standard deviation.

* p < .1. **p < .05. ***p < .01.

The results show that CSR is significantly correlated with risk, with a small overall effect size of *r* = −0.008 (*k* = 101, *N* = 136,360) at *p* < .10, marginally supporting Hypothesis 1. A deeper examination of the subgroup analysis reveals more nuanced results, particularly when decomposing CSR and firm risk. Dimensions of CSR and types of risk explain 13.5% and 15%, respectively, of the heterogeneity in the effect sizes, as reflected in the *R*^2^ values. Governance-related CSR mitigates risk (*r* = −0.067, *p* < .05), while environmental CSR and social CSR do not necessarily mitigate risk (*r* = 0.044 and 0.055, respectively). This suggests that CSR’s role in risk mitigation is stronger in governance-related activities than in environmental or social dimensions. Similarly, CSR activities effectively mitigate market risk (*r* = −0.118, *p* < .01) and operational risk (*r* = −0.021, *p* < .10), but are not effective for financial risk (*r* = 0.011, *p* < .05). In fact, CSR may increase financial risk rather than mitigate it. These results challenge the conventional view that CSR universally mitigates all forms of risk (Orlitzky & Benjamin, 2001).

We find that contextual factors play a significant role in explaining the heterogeneity of the results (see Supplemental Appendix Table C1a). For example, the relationship between CSR and firm risk varies by data source and geographic location. CSR shows a small risk-mitigation effect in studies using secondary databases such as KLD, ASSET 4, and Sustainalytics (*r* = −0.009, *k* = 90, *p* < .10), but is associated with higher risk in survey-based studies (*r* = 0.088, *k* = 11, *p* < .05), highlighting the influence of measurement strategies. Geographically, CSR’s risk-mitigation effect is evident in the United States (*r* = −0.013, *k* = 78, *p* < .05), but CSR may exhibit a burden effect in other countries, where it may even increase risk (*r* = 0.079, *k* = 11, *p* < .01). Additionally, CSR’s risk-mitigation effect is observed in domestic firms (*r* = −0.003, *k* = 67, *p* < .10) but not in multinational enterprises (MNEs; *r* = 0.002, *k* = 7, *p* < .10). These findings highlight that while CSR generally mitigates market risk, its effects depend on contextual factors such as the data source, geographic location, and firm type.

The results indicate a positive correlation between CSIR and firm risk (*r* = 0.063, *k* = 134, *N* = 167,067, *p* < .01), supporting the risk-generating effect (Hypothesis 2). The decomposition of the ESG dimensions accounts for 8.9% of the heterogeneity, while risk types explain 4.4%. Environmental CSIR significantly increases risk (*r* = 0.078, *p* < .01), with a larger effect size than social or governance CSIR. Subgroup analysis reveals positive correlations between CSIR and financial risk (*r* = 0.063, *p* < .01) and operational risk (*r* = 0.067, *p* < .10), but a negative correlation with market risk (*r* = −0.015, *p* < .05). Moderator analysis identifies geographic location as a significant factor in explaining differences in the effect sizes between U.S. and non-U.S. firms and between developed and developing countries, though the direction of the effect remains consistent. In contrast to the CSR-risk relationship, the CSIR-risk relationship is more consistent across contexts. Overall, and consistent with Hypothesis 2, CSIR generates risk, except in the case of market risk, which shows a negative correlation with CSIR.

Table 2 also shows a significant positive correlation between CSR and CSIR (*r* = 0.258, *k* = 145, *N* = 144,274, *p* < .01). While CSP decomposition, geographic location, and data sources significantly moderate this relationship, most subgroup effect sizes remain significantly positive. These results support Hypothesis 3 (i.e., the co-existence of CSR and CSIR), highlighting the role of indirect paths in CSR-CSIR linkage.

**Figure 2.**
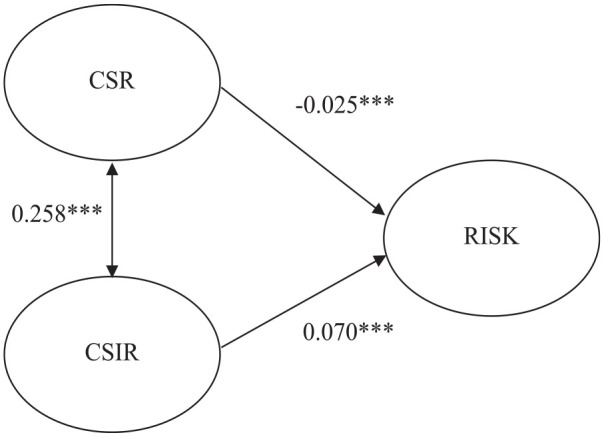
MASEM Results. *Note*. MASEM = meta-analytic structural equation modeling.

Using effect sizes and observation sizes from Table 2 as input, the MASEM analysis results in Table 3 and conceptually illustrated in Figure 2 show that CSR has a significant direct risk-mitigating effect (β = −.025, *p* < .001), which is stronger than the total effect of −0.008. The indirect effect, mediated through CSIR (CSR-CSIR linkage: 0.258 × CSIR-risk linkage: 0.070), is 0.018 and statistically significant at *p* < .001, supporting Hypothesis 4. This suggests that while CSR directly mitigates risk, its positive association with CSIR creates an indirect, risk-increasing pathway, thereby weakening the total effect through a mediating mechanism.

**Table 3.** MASEM Regression Analysis Results.

Coefficients	Model 1	Model 2	Model 3 (CSR + CSIR)	
			Direct effects	Indirect effects
CSR	−.008* (.01)		−.025 (.003)***	.018 (.007)***
CSIR		.063*** (.01)	.070 (.003)***	0 (No path)
Sample size	136,360	167,067	148,145	
*R* ^2^	.004	.067	.068	

*Note*. ***If *p* < .001, ***if *p* < .01, *if *p* < 0.05. OIM standard errors are in brackets. Coefficients in Models 1 and 2 are the same as the main-effect sizes in Table 2. They are listed here in parallel with Model 3 for easy comparison. CSIR = corporate social irresponsibility; CSR = corporate social responsibility; MASEM = meta-analytic structural equation modeling; OIM = observed information matrix.

However, for Hypothesis 5, there is no significant indirect path showing CSR’s influence on CSIR’s positive relationship with firm risk (CSIR–CSR linkage: 0.258 × CSR-risk linkage: −0.025). CSIR’s risk-generating effects remain relatively stable, even when indirect paths are considered. Therefore, Hypothesis 5 is not supported. Overall, while indirect paths through CSIR diminish the risk-mitigation benefits of CSR, no significant indirect effect of CSR was found to reduce CSIR’s risk-generating costs.

We conducted additional analyses to ensure the robustness of our findings, following prior recommendations (Geyskens et al., 2009). These analyses were performed using two different sample sizes—based on the number of firms and observations—to account for differences between longitudinal and cross-sectional data, yielding consistent results (see Supplemental Appendix C: Table C1). Additional tests included removing outliers, applying random-effects meta-analysis recommended by Borenstein et al. (2011), and following the guidelines of Gonzalez-Mulé and Aguinis (2018) for moderator analysis. We also conducted the MASEM for different contingent scenarios using the effect sizes for different subgroups in Table 2 and Supplemental Appendix C to explore the boundary conditions for the dynamic relationships between CSR, CSIR, and risk (see Supplemental Appendix Table C2). Overall, the results are robust, as can be seen in Supplemental Appendix C.

## Discussion

In synthesizing 197 studies, we find that while CSR modestly reduces risk, CSIR’s risk-enhancing effects are stronger, highlighting an asymmetrical pattern. The interplay between CSR and CSIR suggests that they may act as mutual boundary conditions through indirect paths. CSP’s diverse impact on risk across dimensions and contexts underscores this complexity, enhancing our understanding while revealing opportunities for future research. Based on our meta-analysis findings, we identify four key avenues for future research on the CSP–firm risk relationship.

First, future research could investigate the theoretical mechanisms underlying the varied effects of CSP on different types of risk. The differing impacts of CSR and CSIR on market, operational, and financial risks point to the influence of contextual factors, such as institutional settings and stakeholder expectations. Studies could explore how payoff periods vary across risk types and incorporate non-linear or moderated relationships to refine theoretical and practical insights (Fu et al., 2022).

Second, future work should also consider how institutional environments (Marano & Kostova, 2016)—such as stakeholder- versus shareholder-oriented governance systems—moderate the effectiveness of CSR and CSIR. Comparative analyses across U.S. and non-U.S. firms, as well as between domestic firms and MNEs, could illuminate how regional variations and access to CSR/CSIR information shape risk outcomes.

Third, expanding the focus beyond shareholder-relevant risks could enrich our understanding of CSP’s broader implications. Future research might include the risks perceived by employees, customers, or other stakeholders—such as relational, reputational, or litigation risks. Investigating the discrepancies between actual and perceived CSR/CSIR, along with improved measurement approaches, could offer a more comprehensive view of how diverse stakeholder perspectives inform risk assessments.

Lastly, to overcome the limitations of meta-analytic techniques, future research could conduct quasi-experiments or longitudinal studies to establish causality in CSP-risk relationships. In addition, qualitative or mixed-methods approaches may help uncover contextual nuances—such as how stakeholder narratives shape the effect of CSR or CSIR on risk perception. These avenues offer valuable directions for expanding our understanding of CSP’s multifaceted impact on risk across diverse settings and stakeholders.

### Theoretical Contributions

This study makes several important contributions. First, our study makes a theoretical contribution by providing a systematic and comprehensive synthesis of the relationship between CSP and firm risk. This forest-level perspective moves beyond the fragmented, context-specific findings of individual studies, enabling a more integrative understanding of CSP-risk dynamics. In particular, we demonstrate that the effects of CSP are far from uniform: *not all CSP activities equally influence firm risks*. By challenging the common assumption that CSP universally mitigates firm risk, our analysis advances our theoretical understanding by revealing that the risk implications of both CSR and CSIR actions are differentiated and complex. This underscores the need for future research to engage in more nuanced theorization and to pay attention to contextual contingencies.

Second, our research adds to the literature by revealing an asymmetric pattern in which CSIR’s risk-generating effects outweigh CSR’s risk-mitigation effects. This directly addresses the key theoretical question of which effect is greater, a topic that has been largely overlooked. While prior studies have examined CSR and CSIR separately (Kölbel et al., 2017; Shiu & Yang, 2017), few have compared their relative impacts. By analyzing and comparing both linkages, we show that CSIR has a stronger influence on firm risk than CSR. This challenges the assumed symmetry in many theoretical models and calls for greater attention to the differential effects of CSP components. Recognizing this imbalance offers a sharper theoretical lens on how firms manage risk through their social behavior.

Third, our study contributes theoretically by showing a positive correlation between CSR and CSIR and uncovering the indirect pathways through which CSR and CSIR influence firm risk. Failure to account for this linkage may lead to overestimation or underestimation of their effects on firm risks. Most studies (Godfrey et al., 2009; Kölbel et al., 2017) have relied on *bivariate analyses* that examine CSR- or CSIR-outcome relationships, overlooking the interplay between the two constructs. However, our findings reveal that CSR and CSIR are positively correlated, with covarying impacts on risk, indicating that a *multivariate approach*—one that captures indirect paths and complex interrelationships—is more appropriate (Bergh et al., 2016). By employing MASEM, we identify a suppressor effect in which CSR’s risk-mitigating benefits are partially offset by its positive association with CSIR. This challenges the oversimplification of traditional bivariate models and calls for more sophisticated theorizing that accounts for both direct and indirect mechanisms in CSP-risk dynamics.

Lastly, our study makes an important contribution by identifying four key avenues for future research on the CSP–firm risk relationship: theoretical mechanisms, context-specific variations in CSP-risk relationships, advancements in measurement, and the application of advanced methodological approaches. We specifically point out specific areas where further theoretical and empirical work is needed to refine our understanding of how CSP influences firm risk. Examples of relevant research questions in each domain are outlined in Supplemental Appendix D. These directions are crucial for advancing both theoretical development and practical applications in the field of corporate responsibility and risk management.

### Managerial Implications

Our study offers valuable insights for both firms and stakeholders. Firms can use our findings to understand how various dimensions of CSP affect firm risk, emphasizing the need for strategic planning. For example, governance-related CSR significantly reduces risk, while environmental and social CSR are less effective in this regard. These context-dependent findings highlight the importance of strategically prioritizing specific CSP components to effectively manage risks. Moreover, given the asymmetric dynamics of CSR and CSIR, increasing CSR alone is insufficient; minimizing CSIR may have a greater impact. As such, managers should focus on balancing the reduction of CSIR with CSR improvements to enhance a firm’s long-term value. Additionally, stakeholders should recognize that CSR and CSIR are distinct yet interrelated concepts. Since a firm’s CSR and CSIR interact with each other and influence organizational outcomes, stakeholders need to critically assess firm behavior and associated risks.

## Conclusion

How does CSP influence firm risk in today’s business environment, where attention to CSP is increasing, uncertainty is rising, and grand challenges such as the climate crisis are intensifying? The answer remains uncertain. To answer this question, we uncovered a nuanced and complex relationship between CSR, CSIR, and firm risk in this study. Our findings call for a more refined understanding of CSP and offer valuable insights to guide both academic inquiry and strategic decision-making.

## Supplemental Material

sj-docx-1-bas-10.1177_00076503251350044 – Supplemental material for Buffering or Backfiring? A Meta-Analysis of the Effects of Corporate Social (Ir)responsibility on Firm Risk Supplemental material, sj-docx-1-bas-10.1177_00076503251350044 for Buffering or Backfiring? A Meta-Analysis of the Effects of Corporate Social (Ir)responsibility on Firm Risk by Won-Yong Oh, Rong (Ratchel) Zeng and Maoliang Bu in Business & Society

## Notes

1. Despite previous efforts to distinguish CSP from CSR, many empirical studies still use these concepts interchangeably or subsume one under the other (Margolis et al., 2009). To advance the field, our study adopts definitions that align with more empirical operationalizations.
