# Supplementary material for: Buffering or Backfiring? A Meta-Analysis of the Effects of Corporate Social (Ir)responsibility on Firm Risk
Source: Bus Soc. 2025 Jun 29;65(7):1896–915. doi: 10.1177/00076503251350044 (PMC13384389; doi:10.1177/00076503251350044)
Supplement: sj-docx-1-bas-10.1177_00076503251350044 – Supplemental material for Buffering or Backfiring? A Meta-Analysis of the Effects of Corporate Social (Ir)responsibility on Firm Risk [file sj-docx-1-bas-10.1177_00076503251350044.docx]

ONLINE SUPPLEMENTARY MATERIALS

**Appendix A Literature Screening and Inclusion Procedures**

**Literature search**

To ensure a comprehensive search, we followed the following steps:

1. We used search terms such as *corporate social responsibility / CSR, corporate social irresponsibility / CSIR, concern / negative / irresponsib*, unethical corporate activity, corporate culpability, illegal corporate behavior, corporate wrongdoing,* and so on to search databases including Web of Science, ABI/INFORM, JSTOR, Business Source Complete, and Google Scholar.
2. We conducted forward and backward citation searches for several seminal articles (e.g., Kotchen & Moon, 2012; Lange & Washburn, 2012; Strike et al., 2006).
3. We searched journals focused on CSP and cross-referenced datasets from previous meta-analytic reviews of CSR and CSIR (e.g., Frooman, 1997; Hou, Liu, Fan, & Wei, 2016; Orlitzky & Benjamin, 2001; Rost & Ehrmann, 2017).

Our search generated more than 3,000 papers to review for possible inclusion in our meta-analysis.

**Literature screening**

During the screening stage, we carefully reviewed abstracts to identify studies related to a firm’s CSIR engagement. We then skimmed the full texts, including only articles that employed quantitative empirical methods to examine the CSIR phenomenon. This process reduced the total number of papers to 604.

In the coding stage, we carefully analyzed the methodology sections of the screened articles. We included only studies that did not collapse CSR and CSIR into a composite, as our meta-analysis assumes that CSR and CSIR play distinct roles in mitigating or generating firm risk. As a result, papers that operationalized CSR as the difference between CSR and CSIR (e.g., KLD strengths minus weaknesses) were excluded (e.g., Cai et al., 2012; Jo & Na, 2012). We included only studies that provided correlations between CSR and CSIR or between CSR/CSIR and risk. For studies lacking complete correlation information, we contacted the authors to obtain full correlation matrices, which resulted in the inclusion of two additional papers.

Ultimately, our final meta-analysis dataset included 181 papers comprising 197 studies (some papers contained multiple empirical studies), covering a total sample of 222,845 firms and 1,285,635 observations. The list of papers included is provided in Online Appendix E. Our sample represents the most comprehensive collection of studies to date, exceeding tenfold the number included in previous meta-analytic reviews of CSP and risk (Orlitzky & Benjamin, 2001).

**Coding**

We developed a coding platform and protocol (Lipsey & Wilson, 2001) to extract data on all relevant variables and study characteristics. Two research assistants were hired and trained to assist with the process. The corresponding author and the research assistants coded data from 15 articles and achieved a high inter-coder agreement rate of 93.5% (Orwin & Vevea, 2009).

Discrepancies primarily arose from subjective judgments about whether specific variables needed to be coded, but there was no disagreement on extracting effect sizes for key relationships involving CSR and CSIR. During weekly meetings, the research assistants cross-checked their coding results and consulted the corresponding author to resolve any uncertainties.

**Appendix B Meta-Analytic Procedures**

We adopted established methods (Schmidt & Hunter, 2015) and followed the approaches recommended for a rigorous meta-analysis (Geyskens, Krishnan, Steenkamp, & Cunha, 2009).

**Operationalization of Constructs**

We included two independent variables: CSR and CSIR. To analyze these variables comprehensively, we decomposed them into E-, S-, and G- related dimensions.

This approach, which views CSP as a multidimensional construct, aligns with the perspective that firms address various responsibilities across different areas (Margolis, Elfenbein, & Walsh, 2009). While some studies focus on a single dimension (E, S, or G), others examine two or all three domains. To facilitate meaningful comparisons in the subgroup analysis, we coded these dimensions separately. Additionally, we aggregated the dimensions by using the Schmidt and Hunter (2015) method, which allowed us to calculate a composite correlation coefficient for CSR and CSIR based on the correlations among the dimensions reported in the original studies.^[[1]](#footnote-1)^ Following the standard practices in meta-analyses of main effects (Steel, Schmidt, & Shultz, 2008), we used the composite correlation coefficients in the main effect analysis.

The dependent variable, firm risk, is subdivided into three types: market risk, financial risk, and operational risk. We coded these risk types based on the conceptualizations used in prior studies. Consistent with standard practices in meta-analysis, we aggregated all types of risk for the main effect analysis while also coding them separately to enable subgroup moderator analysis (Steel et al., 2008). This aggregation increased the sample size and enhanced the statistical power of the relationships between overall CSR/CSIR and risk. However, it also introduced greater variance in the relationships. To address this commensurability issue, specifically the trade-off between statistical power and the variance inherent in meta-analyses, we conducted both moderator and subgroup analyses (Steel et al., 2008).

As noted in prior research, CSR and CSIR are often operationalized in varying ways. In some cases, different authors have used the same term to represent distinct concepts. For example, some authors have defined CSR as aligning with the definition provided above (e.g., the sum of CSR strengths in the database) (e.g., Strike et al., 2006). In contrast, others have used CSR to refer to the combination of CSR and CSIR (e.g., the sum of strengths minus the sum of concerns in the database) (e.g., Cai et al., 2012; Jo & Na, 2012). To ensure consistency, we carefully compared how these constructs were operationalized across studies and aligned them with the definitions in our framework. A summary of the key variables used in our meta-analysis and their descriptions is provided in Table B1.

**Meta-Analytic Procedures**

When multiple indicators of the same variable were reported in a study, we followed the Schmidt and Hunter (2015) method to calculate a composite index based on the inter-correlation information between these indicators from the original studies. We also coded for sampling and methodological heterogeneity across studies.

Following the Schmidt and Hunter (2015) method, we estimated all coefficients, standard errors, and other statistics for the meta-analyzed pairwise relationships. We applied random-effects estimation methods to calculate these statistics, following established standards in meta-analytic literature (Geyskens et al., 2009; Schmidt & Hunter, 2015).

For the moderator analysis, we employed weighted least squares regression, as it is robust against multicollinearity and heteroscedasticity. In this approach, the weights for the multiple regression analyses were derived from the inverse of the sampling errors (Steel & Kammeyer-Mueller, 2002).

**MASEM: Meta-Analytic Structural Equation Modeling**

Furthermore, we conducted multiple regressions based on MASEM (meta-analytic structural equation modeling), a technique that combines meta-analysis (MA) and structural equation modeling (SEM). This approach is particularly insightful for extending or advancing theoretical frameworks in meta-analysis (Landis, 2013; Viswesvaran & Ones, 1995). We applied it to explore and synthesize the relative impact of CSR and CSIR on risk.

In the first stage, we used the effect sizes between CSR and CSIR, CSR and risk, and CSIR and risk—generated from our meta-analysis—as the correlation inputs required for MASEM. This is one of the recommended approaches for MASEM (Landis, 2013). We then calculated harmonic means based on sample sizes across different correlations and used them to perform MASEM. This approach, considered both defensible and conservative, helps limit the influence of very large values (Landis, 2013; Steel et al., 2008).

In the second stage, we conducted MASEM using the correlations from the first stage as input for the SEM in STATA (v. 15). This allowed us to determine the direct and indirect effects of CSR and CSIR on risk.

Table B1. Key Constructs, Measures, and Study Examples

| **Constructs** | **Examples of Measures** | **Examples of Studies** | |
| --- | --- | --- | --- |
| *CSR* | Index as the sum of all scores for indicators identified as either CSR, CSP strength, or CSR strength;  weighted average of sub-scores for CSP or CSR strengths across different dimensions;  positive activities that may help firms achieve CSR goals. | Cullinan et al. (2016); Godfrey et al. (2009); Mattingly & Berman (2006); Fu et al. (2020); Nguyen & Nguyen (2015); Oh et al. (2017);  Chen et al. (2022); Bouslah et al. (2018); McGuire et al. (2012); Tang & Tang (2016);  Jia & Zhang (2015); Qian (2014). | |
| Environmental CSR | CSP or CSR strengths in environmental dimensions; environmental investments;  weighted average of sub-scores for CSP or CSR strengths across environmental dimensions. | Abeysekera & Fernando (2020); Cullinan et al. (2016); Chung et al. (2012);  Glass et al. (2016); Tang & Tang (2016). | |
| Social CSR | CSP or CSR strengths in social dimensions;  weighted average of sub-scores for CSP or CSR strengths across social dimensions;  investments in community, diversity, employees, or social policies. | Cullinan et al. (2016); Du (2015); Fiaschi et al. (2016); Gregory et al. (2014); Mattingly & Berman (2006);  Harrison & Berman (2016); McGuire et al. (2012); O'Sullivan et al. (2021);  Jia & Zhang (2015). | |
| Governance CSR | CSP or CSR strengths in governance dimensions;  weighted average of sub-scores for CSP or CSR strengths across governance dimensions. | Cullinan et al. (2016); Huseynov & Klamm (2012); Mattingly & Berman (2006). | |
| *CSIR* | Accumulated number of CSIR events, wrongdoing, misconduct, or controversies in which a firm has been involved;  index as the sum of scores for CSP weaknesses or CSP concerns;  weighted average of sub-scores for CSP or CSR weaknesses or concerns across different dimensions;  engagement in irresponsible activities such as corruption. | Bansal (2005); Groening & Kanuri (2018);  Teng & Yang (2021); Zavyalova et al. (2016).  Chen et al. (2022); Cullinan et al. (2016); Nguyen & & Nguyen (2015); Tang & Tang (2016);  Bouslah et al. (2018); McGuire et al. (2012).  Connelly et al. (2022); McKendall & Wagner (1997). | |
| Environmental CSIR | Concerns or weaknesses in environmental issues;  environmental misconduct;  pollution; hazardous waste; total emissions. | Abeysekera & Fernando (2020); Chung et al. (2012); Glass et al. (2016); Post et al. (2011)‘  Du (2015); Qian (2014);  Tang & Tang (2016). | |
| Social CSIR | Concerns or weaknesses in social issues, such as community, diversity, and employees; human rights abuse controversies or events. | Cullinan et al. (2016); Fiaschi et al. (2016); Gregory et al. (2014); Guiliani (2018); Harrison & Berman (2016); Huseynov & Klamm (2012); Mattingly & Berman (2006); McGuire et al. (2012). | |
| Governance CSIR | Concerns or weaknesses in governance issues;  tax avoidance proxies/measures;  CSRC enforcement actions. | Mattingly & Berman (2006)’  Chircop et al. (2018); Zeng (2019)’  Dong et al. (2018). | |
| *Risk* | All types of risk, such as market risk, financial risk, or operational risk, or composites of different types of risk. |  | |
| Market risk | Idiosyncratic risk, as reflected in  fluctuations of stock prices, credit default swaps (traded derivatives);  variation/volatility of daily stock price;   modelled stock responses. | Kölbel et al. (2017);  Mattingly & Berman (2006); Price & Sun (2017); Strike et al. (2006);  Mishra et al. (2013). | |
| Financial risk | Risk from the inability to pay debt, as reflected in  the ratio of total liabilities to total tangible assets;  debt to asset ratio;   debt to equity. | Du (2015); Lee et al. (2018); Shi et al. (2020);  Bouslah et al. (2018); Kotchen & Moon (2012); Oh et al. (2017);  Tang & Tang (2015); Zyglidopoulos et al. (2012). | |
| Operational risk | Risk from fluctuations of future expected cash flows, operations, as reflected in volatility of ROE;  cash volatility;  operational leverage. | Fiaschi et al. (2016); McGuire et al. (2019);  Price & Sun (2017);  Harjoto (2017). | |
| *Sample Characteristics* | |  |  |
| Data source | Database=1, if the study uses archival data from databases such as KLD, ASSET 4, Sustainalytics, etc.  Database=0, if the study uses non-archival data such as interviews and questionnaires. | McGuire et al. (2019); Oh et al. (2017); Walker et al. (2006);  Ding & Wu (2014); Du (2015); Iriyama et al. (2016); Tang & Tang (2016). |  |
| Publication time | Publication year =1, if the publication year is before 2010.  Publication year =0, if the publication year is during or after 2010. |  |  |
| Firm type | Non-MNE=0, if all the sampled firms are domestic firms.  MNE=1, if all the sampled firms are MNEs. | Kim et al. (2018); Ding & Wu (2014); Tang et al. (2015); Zyglidopoulos et al. (2012);  Bu et al. (2016); Fiaschi et al. (2016); Keig et al. (2015); Strike et al. (2006). |  |
| Geographical location | US=1, if the sampled firms are located in the U.S.  US=0, if the sampled firms are not located in the U.S. | Ding & Wu (2014); Kim et al. (2018); Kotchen & Moon (2012); Zyglidopoulos et al. (2012);  Dong et al. (2018); Du (2015); Jia & Zhang (2015). |  |

Please refer to Appendix F for detailed reference information of these papers.

**Appendix C Detailed Tests Results**

**Table C1.** Meta-analytic results including samples based on the number of observations and more subgroup analysis

|  | *K* | *N_1_* | *N_2_* | *r_1_* | *SD_rl_* | *r_2_* | *SD_r2_* | *Q* | *R^2^* | *F* | *90% Cr. I_1_* | *90% Cr. I_2_* |
| --- | --- | --- | --- | --- | --- | --- | --- | --- | --- | --- | --- | --- |
| CSR - Risk | 101 | 136,360 | 556,879 | -0.008^*^ | 0.069 | -0.009^*^ | 0.068 | 591.88 |  |  | [-0.11,0.10] | [-0.12,0.11] |
| **Data source** |  |  |  |  |  |  |  |  | 0.096 | 7.50^*^ |  |  |
| Database | 90 | 111,290 | 390,475 | -0.009^**^ | 0.068 | -0.004 | 0.070 | 560.13 |  |  | [-0.11,0.10] | [-0.12,0.11] |
| Other | 11 | 25,070 | 166,404 | 0.088^**^ | 0.102 | 0.074^***^ | 0.104 | 266.37 |  |  | [-0.03,0.30] | [-0.03,0.30] |
| **Time of publication** | |  |  |  |  |  |  |  | 0.001 | 0.78 |  |  |
| Before 2015 | 37 | 35,923 | 131,000 | 0.007 | 0.087 | -0.003 | 0.082 | 345.58 |  |  | [-0.13,0.14] | [-0.14,0.13] |
| 2015 to 2018 | 39 | 40,744 | 238,762 | -0.024 | 0.074 | -0.021^**^ | 0.079 | 248.79 |  |  | [-0.14,0.09] | [-0.15,0.11] |
| 2018 or after | 25 | 29,693 | 187,117 | 0.008 | 0.044 | 0.010^*^ | 0.041 | 58.37 |  |  | [-0.05,0.06] | [-0.05,0.07] |
| **Firm type** |  |  |  |  |  |  |  |  | 0.002 | 0.01 |  |  |
| Domestic | 67 | 77,901 | 439,656 | -0.003^*^ | 0.068 | -0.006^*^ | 0.060 | 348.90 |  |  | [-0.10,0.10] | [-0.10,0.09] |
| MNE | 7 | 6,460 | 31,902 | 0.002 | 0.085 | -0.042 | 0.130 | 47.62 |  |  | [-0.13,0.13] | [-0.26,0.17] |
| **Geographical location** | |  |  |  |  |  |  |  | 0.090 | 3.91^*^ |  |  |
| U.S. | 78 | 86,499 | 447,215 | -0.013^**^ | 0.070 | -0.009^*^ | 0.067 | 436.04 |  |  | [-0.11,0.09] | [-0.12,0.10] |
| Non-U.S. | 11 | 5,682 | 22,694 | 0.079^***^ | 0.097 | 0.034^*^ | 0.127 | 105.78 |  |  | [-0.06,0.14] | [-0.07,0.14] |
| Developed | 83 | 86,954 | 451,168 | -0.013^**^ | 0.071 | -0.009^*^ | 0.068 | 437.67 | 0.088 | 3.69^*^ | [-0.12,0.09] | [-0.12,0.10] |
| Developing | 6 | 5,227 | 18,741 | 0.082^**^ | 0.093 | 0.027 | 0.074 | 60.97 |  |  | [-0.09,0.25] | [-0.09,0.15] |
| **Type of CSR** |  |  |  |  |  |  |  |  | 0.135 | 18.33^***^ |  |  |
| Environmental | 11 | 6,735 | 31,929 | 0.044^***^ | 0.056 | 0.047^***^ | 0.053 | 21.27 |  |  | [-0.02,0.11] | [-0.03,0.13] |
| Social | 11 | 13,934 | 67,926 | 0.055^**^ | 0.096 | 0.057^***^ | 0.074 | 134.03 |  |  | [-0.10,0.21] | [-0.06,0.18] |
| Governance | 4 | 1,509 | 6,682 | -0.067^**^ | 0.096 | -0.056^***^ | 0.078 | 6.97 |  |  | [-0.14,0.01] | [-0.10,-0.01] |
| **Type of risk** |  |  |  |  |  |  |  |  | 0.150 | 7.88^***^ |  |  |
| Market | 16 | 10,245 | 79,759 | -0.118^***^ | 0.089 | -0.128^***^ | 0.087 | 81.79 |  |  | [-0.25,0.01] | [-0.27,0.01] |
| Financial | 96 | 111,121 | 574,740 | 0.011^**^ | 0.069 | 0.014^***^ | 0.063 | 535 |  |  | [-0.09,0.11] | [-0.09,0.12] |
| Operational | 7 | 3,415 | 16,615 | -0.021^*^ | 0.036 | -0.012^*^ | 0.047 | 4.37 |  |  | [-0.02,0.02] | [-0.08,0.06] |

**Table C1a: CSR-Risk**

p<0.1 *; p <0.05 **; p <0.01 ***; *k:* number of studies; *N_1_:* sample size in number of firms; *N_2_:* sample size in number of observations; *r_1_:* weighted and reliability corrected correlation when using *N_1_*; *SD_r1_:* standard deviation when using *N_1_*; *r_2_:* weighted and reliability corrected correlation when using *N_2_*; *SD_r2_:* standard deviation when using *N_2_*; Cr. I*_1:_* credibility interval when using *N_1;_* Cr. I*_2:_* credibility interval when using *N_2._*

|  | *K* | *N_1_* | *N_2_* | *r_1_* | *SD_rl_* | *r_2_* | *SD_r2_* | *Q* | *R^2^* | *F* | *90% Cr. I_1_* | *90% Cr. I_2_* |
| --- | --- | --- | --- | --- | --- | --- | --- | --- | --- | --- | --- | --- |
| CSIR - Risk | 134 | 167,067 | 929,941 | 0.063^***^ | 0.078 | 0.060^***^ | 0.073 | 1,031.71 |  |  | [-0.06,0.18] | [-0.06,0.18] |
| **Data source** |  |  |  |  |  |  |  |  | 0.04 | 7.00^***^ |  |  |
| Database | 123 | 140,939 | 776,371 | 0.059^***^ | 0.072 | 0.057^***^ | 0.065 | 745.72 |  |  | [-0.05,0.17] | [-0.05,0.16] |
| Other | 11 | 26,128 | 153,570 | 0.082^***^ | 0.109 | 0.078^***^ | 0.105 | 317.56 |  |  | [-0.09,0.26] | [-0.09,0.25] |
| **Time of publication** | |  |  |  |  |  |  |  | 0.073 | 6.00^***^ |  |  |
| Before 2015 | 45 | 42,016 | 153,530 | 0.080^***^ | 0.088 | 0.084^***^ | 0.079 | 334.17 |  |  | [-0.05,0.21] | [-0.04,0.21] |
| 2015 to 2018 | 48 | 57,484 | 335,591 | 0.087^***^ | 0.085 | 0.084^***^ | 0.087 | 449.48 |  |  | [-0.05,0.22] | [-0.05,0.22] |
| 2018 or after | 41 | 67,567 | 40,820 | 0.032^***^ | 0.049 | 0.033^***^ | 0.051 | 160.80 |  |  | [-0.04,0.10] | [-0.05,0.12] |
| **Firm type** |  |  |  |  |  |  |  |  | 0.008 | 0.70 |  |  |
| Domestic | 93 | 121,676 | 668,355 | 0.073^***^ | 0.074 | 0.071^***^ | 0.072 | 684.55 |  |  | [-0.04,0.19] | [-0.05,0.19] |
| MNE | 9 | 12,571 | 64,194 | 0.051^**^ | 0.071 | 0.024 | 0.082 | 64.54 |  |  | [-0.06,0.16] | [-0.11,0.16] |
| **Geographical location** | |  |  |  |  |  |  |  | 0.056 | 5.51^***^ |  |  |
| U.S. | 92 | 94,120 | 513,456 | 0.071^***^ | 0.079 | 0.068^***^ | 0.067 | 599.79 |  |  | [-0.05,0.19] | [-0.04,0.18] |
| Non-U.S. | 29 | 39,588 | 192,267 | 0.037^***^ | 0.050 | 0.031^***^ | 0.050 | 99.58 |  |  | [-0.03,0.11] | [-0.05,0.11] |
| Developed | 99 | 100,942 | 543,727 | 0.068^***^ | 0.077 | 0.066^***^ | 0.066 | 609.04 | 0.040 | 3.85^***^ | [-0.05,0.18] | [-0.04,0.17] |
| Developing | 22 | 32,766 | 161,996 | 0.038^***^ | 0.055 | 0.030^***^ | 0.065 | 100.54 |  |  | [-0.04,0.12] | [-0.06,0.12] |
| **Type of CSIR** |  |  |  |  |  |  |  |  | 0.089 | 2.48^*^ |  |  |
| Environmental | 14 | 11,493 | 43,824 | 0.078^***^ | 0.089 | 0.099^***^ | 0.081 | 94.97 |  |  | [-0.06,0.21] | [-0.03,0.23] |
| Social | 14 | 16,025 | 65,618 | 0.057^***^ | 0.063 | 0.063^***^ | 0.063 | 63.20 |  |  | [-0.03,0.15] | [-0.04,0.16] |
| Governance | 15 | 22,736 | 119,893 | 0.051^***^ | 0.048 | 0.055^***^ | 0.044 | 52.22 |  |  | [-0.01,0.12] | [-0.02,0.13] |
| **Type of risk** |  |  |  |  |  |  |  |  | 0.044 | 3.35^***^ |  |  |
| Market | 21 | 15,693 | 119,177 | -0.015^**^ | 0.071 | -0.017^*^ | 0.072 | 63.59 |  |  | [-0.11,0.08] | [-0.13,0.10] |
| Financial | 132 | 168,198 | 928,199 | 0.063^***^ | 0.079 | 0.060^***^ | 0.077 | 1,115.56 |  |  | [-0.06,0.19] | [-0.07,0.19] |
| Operational | 8 | 6,036 | 26,847 | 0.067^*^ | 0.098 | 0.066^*^ | 0.103 | 59.23 |  |  | [-0.08,0.22] | [-0.10,0.23] |

**Table C1b: CSIR-Risk**

p<0.1 *; p <0.05 **; p <0.01 ***; *k:* number of studies; *N_1_:* sample size in number of firms; *N_2_:* sample size in number of observations; *r_1_:* weighted and reliability corrected correlation when using *N_1_*; *SD_r1_:* standard deviation when using *N_1_*; *r_2_:* weighted and reliability corrected correlation when using *N_2_*; *SD_r2_:* standard deviation when using *N_2_*; Cr. I*_1:_* credibility interval when using *N_1;_* Cr. I*_2:_* credibility interval when using *N_2._*

|  | *K* | *N_1_* | *N_2_* | *r_1_* | *SD_rl_* | *r_2_* | *SD_r2_* | *Q* | *R^2^* | *F* | *90% Cr. I_1_* | *90% Cr. I_2_* |
| --- | --- | --- | --- | --- | --- | --- | --- | --- | --- | --- | --- | --- |
| CSR - CSIR | 145 | 144,274 | 815,706 | 0.258^***^ | 0.156 | 0.265^***^ | 0.148 | 4,183.33 |  |  | [0.01,0.51] | [0.02,0.51] |
| **Data source** |  |  |  |  |  |  |  |  | 0.057 | 43.02^***^ |  |  |
| Database | 135 | 137,217 | 795,051 | 0.273^***^ | 0.149 | 0.272^***^ | 0.144 | 3683.15 |  |  | [0.03,0.51] | [0.04,0.51] |
| Other | 10 | 7,057 | 20,655 | 0.027^*^ | 0.105 | -0.007 | 0.104 | 83.39 |  |  | [-0.14,0.19] | [-0.17,0.16] |
| **Time of publication** | |  |  |  |  |  |  |  | 0.006 | 0.64 |  |  |
| Before 2015 | 60 | 49,817 | 221,622 | 0.254^***^ | 0.135 | 0.243^***^ | 0.142 | 1,045.89 |  |  | [0.04,0.47] | [0.01,0.47] |
| 2015 to 2018 | 51 | 55,754 | 356,738 | 0.292^***^ | 0.112 | 0.308^***^ | 0.097 | 781.25 |  |  | [0.11,0.47] | [0.13,0.48] |
| 2018 or after | 34 | 38,703 | 237,346 | 0.196^***^ | 0.201 | 0.222^***^ | 0.163 | 1896.44 |  |  | [-0.03,0.50] | [-0.05,0.49] |
| **Firm type** |  |  |  |  |  |  |  |  | 0.003 | 0.18 |  |  |
| Domestic | 87 | 110,708 | 584,509 | 0.267^***^ | 0.172 | 0.269^***^ | 0.152 | 4,043.89 |  |  | [-0.01,0.55] | [0.02,0.52] |
| MNE | 9 | 7,239 | 33,026 | 0.209^**^ | 0.150 | 0.229^***^ | 0.127 | 186.69 |  |  | [-0.03,0.45] | [0.02,0.44] |
| **Geographical location** | |  |  |  |  |  |  |  |  |  |  |  |
| U.S. | 114 | 117,223 | 635,655 | 0.286^***^ | 0.155 | 0.285^***^ | 0.141 | 3,577.47 | 0.156 | 47.94^***^ | [0.03,0.54] | [0.05,0.52] |
| Non-U.S. | 19 | 12,188 | 56,378 | -0.012 | 0.111 | -0.027 | 0.121 | 157.91 |  |  | [-0.18,0.16] | [-0.22,0.17] |
| Developed | 124 | 118,845 | 641,257 | 0.284^***^ | 0.157 | 0.279^***^ | 0.140 | 3,988.11 | 0.153 | 49.90^***^ | [0.02,0.53] | [0.06,0.51] |
| Developing | 9 | 10,566 | 50,776 | -0.029 | 0.088 | -0.044 | 0.097 | 85.57 |  |  | [-0.17,0.11] | [-0.20,0.11] |
| **Type of CSR/CSIR** |  |  |  |  |  |  |  |  | 0.246 | 11.46^***^ |  |  |
| Environmental | 17 | 8,694 | 46,226 | 0.322^***^ | 0.090 | 0.328^***^ | 0.062 | 85.00 |  |  | [0.19,0.45] | [0.23,0.42] |
| Social | 14 | 12,207 | 55,301 | 0.130^***^ | 0.102 | 0.126^***^ | 0.083 | 135.15 |  |  | [-0.03,0.29] | [-0.01,0.26] |
| Governance | 6 | 2,965 | 14,093 | 0.055 | 0.275 | 0.171 | 0.256 | 259.66 |  |  | [-0.39,0.50] | [-0.25,0.59] |

**Table C1c: CSR and CSIR**

p<0.1 *; p <0.05 **; p <0.01 ***; *k:* number of studies; *N_1_:* sample size in number of firms; *N_2_:* sample size in number of observations; *r_1_:* weighted and reliability corrected correlation when using *N_1_*; *SD_r1_:* standard deviation when using *N_1_*; *r_2_:* weighted and reliability corrected correlation when using *N_2_*; *SD_r2_:* standard deviation when using *N_2_*; Cr. I*_1:_* credibility interval when using *N_1;_* Cr. I*_2:_* credibility interval when using *N_2._*.

Table C2. MASEM regression analysis results – Samples based on the number of observations

| **Coefficients** | Model 4 | Model 5 | Model 6 (CSR+CSIR) | | | |
| --- | --- | --- | --- | --- | --- | --- |
|  |  |  | Direct Effects | | Indirect Effects | |
| **CSR** | -0.009^*^  (0.01) |  | -0.027  (.004) | ^***^ | 0.017  (0.003) | ^***^ |
| **CSIR** |  | 0.060^***^  (0.01) | 0.067  (.004) | ^***^ | 0  (no path) |  |
| **Sample size** | 556,879 | 929,941 | 732,245 | | |  |
| ***R^2^*** | 0.004 | 0.070 | 0.071 | | |  |

*** if p < 0.001, *** if p < 0.01, * if p <0.05. OIM Standard errors are in brackets.

Note: Models 4-6 are based on the number of observations as sample size.

**Appendix D Examples of Research Questions for Future Exploration**

| **Research Opportunity** | **Examples of Research Questions** |
| --- | --- |
| Theoretical mechanisms | - What theoretical mechanisms drive the varying impacts of CSR and CSIR on market, operational, and financial risks? - How do contextual factors, such as institutional settings or stakeholder expectations, influence these mechanisms? - What is the role of timeframes and payoff periods in shaping CSP-risk relationships across risk types? - How can non-linear or moderated relationships enhance our understanding of CSP-risk dynamics? |
| Research context | - How do institutional environments (e.g., stakeholder- vs. shareholder-oriented systems) affect CSR and CSIR efficacy? - Why do U.S.-based firms and domestic firms experience stronger stakeholder responses to CSP activities compared to non-U.S. firms and MNEs? - How do cross-national differences and regional variations in access to CSR/CSIR information impact CSP-risk linkages? - What drives the variation in CSP-risk relationships across firms differing in size, reputation, or resource availability? |
| Measurement | - How can researchers bridge the gaps between actual and perceived CSR/CSIR and their impacts on risk? - What methodologies can capture relational, reputational, and litigation risks in CSP-risk studies? - How do stakeholder-specific perspectives (e.g., employees, customers) refine CSP-risk assessments? - How can the interaction among the E, S, and G dimensions be better measured to understand their combined effects on risk? |
| Research methods | - How can quasi-experimental designs or longitudinal studies establish causality in CSP-risk relationships? - How can qualitative or mixed-methods approaches reveal nuanced relationships in CSP-risk dynamics? - What role can qualitative meta-analyses play in complementing the quantitative findings on CSP and risk? - How can combining qualitative and quantitative methods uncover broader insights into CSP and risk mechanisms? |

**Appendix E Quantitative Papers Included in Our Meta-analysis Dataset**

Abeysekera, A. P., & Fernando, C. S. (2020). Corporate social responsibility versus corporate shareholder responsibility: A family firm perspective. *Journal of Corporate Finance, 61*, 101370.

Afrin, R., Peng, N., & Bowen, F. (2022). The wealth effect of corporate water actions: How past corporate responsibility and irresponsibility influence stock market reactions. *Journal of Business Ethics, 180*(1), 105-124.

Alakent, E., & Ozer, M. (2014). Can companies buy legitimacy? Using corporate political strategies to offset negative corporate social responsibility records. *Journal of Strategy and Management, 7*(4), 318-336.

Arora, P., & Dharwadkar, R. (2011). Corporate governance and corporate social responsibility (CSR), The moderating roles of attainment discrepancy and organization slack. *Corporate Governance: An International Review, 19*(2), 136-152.

Bansal, P. (2005). Evolving sustainably: A longitudinal study of corporate sustainable development. *Strategic Management Journal, 26*(3), 197-218.

Boukattaya, S., & Abdelwahed, O. (2021). Corporate social practices and firm financial performance: Empirical evidence from France. *International Journal of Financial Studies*, 9(4), 54.

Bouslah, K., Linares-Zegarra, J., M'Zali, B., & Scholtens, B. (2018). CEO risk-taking incentives and socially irresponsible activities. *British Accounting Review, 50*(1), 76-92.

Branzei, O., Frooman, J., McKnight, B., & Zietsma, C. (2018). What good does doing good do? The effect of bond rating analysts’ corporate bias on investor reactions to changes in social responsibility. *Journal of Business Ethics, 148*(1), 183-203.

Brower, J., Kashmiri, S., & Mahajan, V. (2017). Signaling virtue: Does firm corporate social performance trajectory moderate the social performance–financial performance relationship? *Journal of Business Research, 81*, 86-95.

Bu, M. L., & Wagner, M. (2016). Racing to the bottom and racing to the top: The crucial role of firm characteristics in foreign direct investment choices. *Journal of International Business Studies, 47*(9), 1032-1057.

Burke, J. J., Hoitash, R., & Hoitash, U. (2019). The heterogeneity of board-level sustainability committees and corporate social performance. *Journal of Business Ethics*, *154*(4), 1161-1186.

Burke, J. J. (2022). Do boards take environmental, social, and governance issues seriously? Evidence from media coverage and CEO dismissals. *Journal of Business Ethics*, *176*, 647–671.

Cajias, M., Fuerst, F., & Bienert, S. (2014). Can investing in corporate social responsibility lower a company's cost of capital? *Studies in Economics and Finance*, *31*(2), 202-222.

Camara, A., & Petrenko, O. (2021). The influence of diversity and employee relations on corporate philanthropy and performance. *Business and Society Review*, *126*(4), 407-431.

Carberry, E. J., Engelen, P.-J., & Van Essen, M. (2018). Which firms get punished for unethical behavior? Explaining variation in stock market reactions to corporate misconduct. *Business Ethics Quarterly, 28*(2), 119-151.

Chang, K., Kim, I., & Li, Y. (2014). The heterogeneous impact of corporate social responsibility activities that target different stakeholders. *Journal of Business Ethics, 125*(2), 211-234.

Chatterji, A. K., Levine, D. I., & Toffel, M. W. (2009). How well do social ratings actually measure corporate social responsibility? *Journal of Economics and Management Strategy, 18*(1), 125-169.

Chen, C.-J., Guo, R.-S., Hsiao, Y.-C., & Chen, K.-L. (2018). How business strategy in non-financial firms moderates the curvilinear effects of corporate social responsibility and irresponsibility on corporate financial performance. *Journal of Business Research*, 92, 154-167.

Chen, D., Chen, Y., Li, O. Z., & Ni, C. (2018). Foreign residency rights and corporate fraud. *Journal of Corporate Finance, 51*, 142-163.

Chen, J. J., Ko, S. I., Li, L. S., & Yang, F. X. (2021). Are better-connected CEOs more socially responsible? Evidence from the US restaurant industry. *Tourism Management, 85*, 104304.

Chen, J., Dong, W., Tong, Y., & Zhang, F. (2020). Corporate philanthropy and corporate misconduct: Evidence from China. *International Review of Economics & Finance, 65*, 17-31.

Chen, S., Hermes, N., & Hooghiemstra, R. (2022). Corporate social responsibility and NGO directors on boards. *Journal of Business Ethics, 175*(3), 625-649.

Chiang, W.-C., Shang, J., & Sun, L. (2017). Broad bond rating change and irresponsible corporate social responsibility activities. *Advances in Accounting, 39*, 32-46.

Chircop, J., Fabrizi, M., Ipino, E., & Parbonetti, A. (2018). Does social capital constrain firms' tax avoidance? *Social Responsibility Journal, 14*(3), 542-565.

Chiu, S.-C., & Sharfman, M. (2018). Corporate social irresponsibility and executive succession: An empirical examination. *Journal of Business Ethics, 149*(3), 707- 723.

Chung, H., Lin, J. R., & Yang, Y. S. (2012). How do entrenched managers handle stakeholders interests? *Journal of Multinational Financial Management, 22*(5), 263-277.

Col, B., & Patel, S. (2019). Going to haven? Corporate social responsibility and tax avoidance. *Journal of Business Ethics, 154*(4), 1033-1050.

Connelly, B. L., Shi, W., & Zyung, J. (2017). Managerial response to constitutional constraints on shareholder power. *Strategic Management Journal, 38*(7), 1499- 1517.

Connelly, B. L., Shi, W., Jack, W. H., & Hersel, M. C. (2022). Searching for a sign: CEO successor selection in the wake of corporate misconduct. *Journal of Management, 48*(4), 1035-1066.

Corciolani, M., Nieri, F., & Tuan, A. (2020). Does involvement in corporate social irresponsibility affect the linguistic features of corporate social responsibility reports? *Corporate Social Responsibility and Environmental Management, 27*(2), 670-680.

Cui, J., Jo, H., & Na, H. (2018). Does corporate social responsibility affect information asymmetry? *Journal of Business Ethics, 148*(3), 549-572.

Cui, V., Ding, S., Liu, M., & Wu, Z. (2018). Revisiting the effect of family involvement on corporate social responsibility: A behavioral agency perspective. *Journal of Business Ethics, 152*(1), 291-309.

Cullinan, C. P., Mahoney, L. S., & Roush, P. (2016). Corporate social responsibility and shareholder support for corporate governance changes. *Social Responsibility Journal, 12*(4), 687-705.

Cumming, D., Leung, T. Y., & Rui, O. (2015). Gender diversity and securities fraud. *Academy of Management Journal*, *58*(5), 1572-1593.

Dang, A., & Nguyen, T. (2021). Valuation effect of emotionality in corporate philanthropy. *Journal of Business Ethics*, *173*(1), 47-67.

Deng, C., Kanagaretnam, K., & Zhou, Z. (2020). Do locally based independent directors reduce corporate misconduct? Evidence from Chinese listed firms. *Journal of International Accounting Research*, 19(3), 61-90.

Dharwadkar, R., Guo, J., Shi, L. N., & Yang, R. (2021). Corporate social irresponsibility and boards: The implications of legal expertise. *Journal of Business Research*, *125*, 143-154.

Doh, J. P., Howton, S. D., Howton, S. W., & Siegel, D. S. (2009). Does the market respond to an endorsement of social responsibility? The role of institutions, information, and legitimacy. *Journal of Management, 36*(6), 1461-1485.

Dong, W., Han, H., Ke, Y., & Chan, K. C. (2018). Social trust and corporate misconduct: Evidence from China. *Journal of Business Ethics*, *151*(2), 539-562.

Drori, R. M. I. (2016). Corporate social action and newspaper media: The role of geopolitical risk. In *Corporate Responsibility* (pp. 117-139), Springer.

Du, X. (2015). Is corporate philanthropy used as environmental misconduct dressing? Evidence from Chinese family-owned firms. *Journal of Business Ethics, 129*(2), 341-361.

Du, X. Q. (2015). How the market values greenwashing? Evidence from China. *Journal of Business Ethics, 128*(3), 547-574.

Feng, Z.-Y., Chen, C. R., & Tseng, Y.-J. (2018). Do capital markets value corporate social responsibility? Evidence from seasoned equity offerings. *Journal of Banking & Finance, 94*, 54-74.

Fiaschi, D., Giuliani, E., & Nieri, F. (2017). Overcoming the liability of origin by doing no-harm: Emerging country firms’ social irresponsibility as they go global. *Journal of World Business, 52*(4), 546-563.

Flammer, C. (2013). Corporate social responsibility and shareholder reaction: The environmental awareness of investors. *Academy of Management Journal, 56*(3), 758-781.

Friske, W., Nikolov, A. N., & Hoang, P. (2020). CSR reporting practices: An integrative model and analysis. Journal of Marketing Theory and Practice, 28(2), 138-155.

Fu, L. (2017). *Uncovering the two faces: Drivers, contexts, and outcomes of corporate social inconsistency.* (PhD), University of Adelaide, Adelaide, South Australia.

Fu, R., Tang, Y., & Chen, G. (2020). Chief sustainability officers and corporate social (ir)responsibility. *Strategic Management Journal*, *41*(4), 656-680.

Fu, L., Boehe, D. M., & Orlitzky, M. O. (2022). Broad or narrow stakeholder management? A signaling theory perspective. *Business & Society*, 61(7): 1838-1880.

Gao, Y., & Yang, H. (2021). Does ownership matter? Firm ownership and corporate illegality in China. *Journal of Business Ethics*, 168(2), 431-445.

Gao, Y. Q., Yang, H. B., & Zhang, M. H. (2021). Too bad to fear, too good to dare? Performance feedback and corporate misconduct. *Journal of Business Research*, *131*, 1-11.

Ge, W., & Liu, M. (2015). Corporate social responsibility and the cost of corporate bonds. *Journal of Accounting and Public Policy*, *34*(6), 597-624.

Giuliani, E., Vezzulli, A., & Nieri, F. (2018). Explaining organizational wrongdoing by emerging country firms. *Academy of Management Proceedings*, *2018*(1), 14144.

Glass, C., Cook, A., & Ingersoll, A. R. (2016). Do women leaders promote sustainability? Analyzing the effect of corporate governance composition on environmental performance. *Business Strategy and the Environment*, *25*(7), 495-511.

Godfrey, P. C., Merrill, C. B., & Hansen, J. M. (2009). The relationship between corporate social responsibility and shareholder value: An empirical test of the risk management hypothesis. *Strategic Management Journal*, *30*(4), 425-445.

Golden, J., Sun, L., & Zhang, J. H. (2017). Corporate social responsibility and goodwill impairment. *Accounting and the Public Interest, 18*(1), 1-28.

Gong, M., Zhang, Z., & Jia, M. (2019). Lie detectors? How entrepreneurs’ facial expressions during IPO roadshow presentations predict new venture misconduct behaviors. *IEEE Transactions on Engineering Management,* 68(6), 1855-1866.

Gregory, A., Tharyan, R., & Whittaker, J. (2014). Corporate social responsibility and firm value: Disaggregating the effects on cash flow, risk and growth. *Journal of Business Ethics, 124*(4), 633-657.

Groening, C., & Kanuri, V. K. (2018). Investor reactions to concurrent positive and negative stakeholder news. *Journal of Business Ethics, 149*(4), 833-856.

Guillamon-Saorin, E., Kapelko, M., & Stefanou, S. E. (2018). Corporate social responsibility and operational inefficiency: A dynamic approach. *Sustainability (Switzerland), 10*(7), 2277.

Gunae, C., Christmann, P., & Tae-Nyun, K. (2015). Target CSR as a signal in acquisitions: Its effect on acquisition premium. *Academy of Management Annual Meeting Proceedings, 2015*(1), 1-1.

Gupta, V. K., Mortal, S., Chakrabarty, B., Guo, X., & Turban, D. B. (2020). CFO gender and financial statement irregularities. *Academy of Management Journal*, *63*(3), 802-831.

Harjoto, M., Laksmana, I., & Lee, R. (2015). Board diversity and corporate social responsibility. *Journal of Business Ethics, 132*(4), 641-660.

Harjoto, M. A. (2017). Corporate social responsibility and degrees of operating and financial leverage. *Review of Quantitative Finance and Accounting, 49*(2), 487- 513.

Harjoto, M. A., & Salas, J. (2017). Strategic and institutional sustainability: Corporate social responsibility, brand value, and Interbrand listing. *Journal of Product and Brand Management, 26*(6), 545-558.

Harrison, J. S., & Berman, S. L. (2016). Corporate social performance and economic cycles. *Journal of Business Ethics, 138*(2), 279-294.

Hasija, D. B., Ellstrand, A. E., Worrell, D. L., & Dixon-Fowler, H. (2017). Two heads may be more responsible than one: Co-CEOs and corporate social performance. *Journal of Management Policy and Practice, 18*(2), 9-21.

Hoi, C. K., Wu, Q., & Zhang, H. (2013). Is corporate social responsibility (CSR) associated with tax avoidance? Evidence from irresponsible CSR activities. *Accounting Review, 88*(6), 2025-2059.

Hoi, C. K., Wu, Q., & Zhang, H. (2018). Community social capital and corporate social responsibility. *Journal of Business Ethics, 152*(3), 647-665.

Hong, J. K., Lee, J. H., & Roh, T. (2022). The effects of CEO narcissism on corporate social responsibility and irresponsibility. *Managerial and Decision Economics*, *43*(6), 1926-1940.

Hsu, A., Koh, K., Liu, S., & Tong, Y. H. (2019). Corporate social responsibility and corporate disclosures: An investigation of investors’ and analysts’ perceptions. *Journal of Business Ethics, 158*(2), 507-534.

Huseynov, F., & Klamm, B. K. (2012). Tax avoidance, tax management and corporate social responsibility. *Journal of Corporate Finance, 18*(4), 804-827.

Jackson, G., Bartosch, J., Avetisyan, E., Kinderman, D., & Jette Steen, K. (2020). Mandatory non-financial disclosure and its influence on CSR: An international comparison. *Journal of Business Ethics, 162*(2), 323-342.

Jain, T., & Zaman, R. (2020). When boards matter: The case of corporate social irresponsibility. *British Journal of Management*, *31*(2), 365-386.

Jeong, N., & Kim, N. (2020). The effects of political orientation on corporate social (ir)responsibility. *Management Decision*, *58*(2), 255-266.

Jeong, N., Kim, N., & Arthurs, J. D. (2021). The CEO’s tenure life cycle, corporate social responsibility and the moderating role of the CEO’s political orientation. *Journal of Business Research*, *137*, 464-474.

Jha, A. (2019). Financial reports and social capital. *Journal of Business Ethics, 155*(2), 567-596.

Jia, M., & Zhang, Z. (2015). News visibility and corporate philanthropic response: Evidence from privately owned Chinese firms following the Wenchuan earthquake. *Journal of Business Ethics, 129*(1), 93-114.

Jung, S., Kim, J. H., Kang, K. H., & Kim, B. (2018). Internationalization and corporate social responsibility in the restaurant industry: Risk perspective. *Journal of Sustainable Tourism, 26*(7), 1105-1123.

Kang, C., Germann, F., & Grewal, R. (2016). Washing away your sins? Corporate social responsibility, corporate social irresponsibility, and firm performance. *Journal of Marketing, 80*(2), 59-79.

Kang, C. A. (2014). *Doing good, doing bad, and doing well: Investigating the dynamic effectiveness of sustainability strategy.* (PhD), Pennsylvania State University, Pennsylvania, USA.

Kang, K. H., Lee, S., & Huh, C. (2010). Impacts of positive and negative corporate social responsibility activities on company performance in the hospitality industry. *International Journal of Hospitality Management, 29*(1), 72-82.

Kanuri, V. K., Houston, R., & Andrews, M. (2020). Firms behaving badly? Investor reactions to corporate social irresponsibility. *Business and Society Review*, 125(1), 41-70.

Karim, K., Lee, E., & Suh, S. (2018). Corporate social responsibility and CEO compensation structure. *Advances in Accounting, 40*, 27-41.

Keig, D. L., Brouthers, L. E., & Marshall, V. B. (2015). Formal and informal corruption environments and multinational enterprise social irresponsibility. *Journal of Management Studies, 52*(1), 89-116.

Kim, B., Moon, J. J., & Kim, E. (2020). Executive migration matters: The transfer of CSR profiles across organizations. *Business & Society*, *61*(1), 155-190.

Kim, K.-H., Kim, M., & Qian, C. (2018). Effects of corporate social responsibility on corporate financial performance: A competitive-action perspective. *Journal of Management, 44*(3), 1097-1118.

King, A., & Lenox, M. (2002). Exploring the locus of profitable pollution reduction. *Management Science*, *48*(2), 289-299.

King, A. A., & Lenox, M. J. (2001). Does it really pay to be green? An empirical study of firm environmental and financial performance: An empirical study of firm environmental and financial performance. *Journal of Industrial Ecology, 5*(1), 105-116.

Kölbel, J. F., Busch, T., & Jancso, L. M. (2017). How media coverage of corporate social irresponsibility increases financial risk. *Strategic Management Journal, 38*(11), 2266-2284.

Kotchen, M., & Moon, J. J. (2012). Corporate social responsibility for irresponsibility. B.E. *Journal of Economic Analysis and Policy*, *12*(1), 1-23.

LaGore, W. D., Mahoney, L. S., & Thorne, L. (2011) Financial restatement, corporate social responsibility, and CEO compensation. In*: Vol. 15. Research on Professional Responsibility and Ethics in Accounting* (pp. 101-126).

Lamb, N. H., Butler, F., & Roundy, P. (2017). Family firms and corporate social responsibility: Exploring “concerns”. *Journal of Strategy and Management,10*(4), 469-487.

Lamb, N. H., & Butler, F. C. (2018). The influence of family firms and institutional owners on corporate social responsibility performance. *Business and Society, 57*(7), 1374-1406.

Lee, C., Palmon, D., & Yezegel, A. (2018). The corporate social responsibility information environment: Examining the value of financial analysts’ recommendations. *Journal of Business Ethics, 150*(1), 279-301.

Lee, D. (2017). Corporate social responsibility and management forecast accuracy. *Journal of Business Ethics*, *140*(2), 353-367.

Lee, K., Oh, W.-Y., & Kim, N. (2013). Social media for socially responsible firms: Analysis of Fortune 500's twitter profiles and their CSR/CSIR ratings. *Journal of Business Ethics, 118*(4), 791-806.

Lenz, I., Wetzel, H., & Hammerschmidt, M. (2017). Can doing good lead to doing poorly? Firm value implications of CSR in the face of CSI. *Journal of the Academy of Marketing Science, 45*(5), 677-697.

Li, J., Haider, Z. A., Jin, X., & Yuan, W. (2019). Corporate controversy, social responsibility and market performance: International evidence. *Journal of International Financial Markets, Institutions and Money, 60*, 1-18.

Li, X., Jeong-Bon, K., Wu, H., & Yu, Y. (2021). Corporate social responsibility and financial fraud: The moderating effects of governance and religiosity. *Journal of Business Ethics*, *170*(3), 557-576.

Lin, H., Zeng, S., Wang, L., Zou, H., & Ma, H. (2016). How does environmental irresponsibility impair corporate reputation? A multi-method investigation. *Corporate Social Responsibility and Environmental Management*, *23*(6), 413-423.

Lin, H.-C., Wang, C.-S., & Wu, R.-S. (2017). Does corporate ethics help investors forecast future earnings? *Social Responsibility Journal*, *13*(1), 62-77.

Liu, C., Wang, S. L., & Li, D. (2022). Hidden in a group? Market reactions to multi‐violator corporate social irresponsibility disclosures. *Strategic Management Journal*, *43*(1), 160-179.

Liu, M., Shi, Y., Wilson, C., & Wu, Z. (2017). Does family involvement explain why corporate social responsibility affects earnings management? *Journal of Business Research, 75*, 8-16.

Liu, Z., Zeng, S., Xu, X., Lin, H., & Ma, H. (2019). Corporate misconduct, trade credit and charitable donations: Evidence from Chinese listed companies. *Chinese Management Studies*, *13*(3), 664-686.

Luo, J., Bi, M., & Jia, D. (2022). Corporate social responsibility risk and firm performance: A network perspective. *International Journal of Financial Studies*, *10*(2), 40.

Mahoney, L. S., & Thorn, L. (2006). An examination of the structure of executive compensation and corporate social responsibility: A Canadian investigation. *Journal of Business Ethics, 69*(2), 149-162.

Mahoney, L. S., & Thorne, L. (2005). Corporate social responsibility and long-term compensation: Evidence from Canada. *Journal of Business Ethics, 57*(3), 241- 253.

Malikov, K., & Gaia, S. (2022). Do CEO social connections promote corporate malpractices? Evidence from classification shifting. *Accounting Forum*, 46(4), 369–393.

Manner, M. H. (2010). The impact of CEO characteristics on corporate social performance. *Journal of Business Ethics*, *93*(Supplement 1), 53-72.

Markoczy, L., Kolev, K. D., & Qian, C. (2022). Trade-off among stakeholders: CEO political orientation and corporate social irresponsibility. *Long Range Planning*, *56*(2), 102273.

Mattingly, J. E., & Berman, S. L. (2006). Measurement of corporate social action: Discovering taxonomy in the Kinder Lydenburg Domini ratings data. *Business & Society, 45*(1), 20-46.

McGuire, J., Dow, S., & Argheyd, K. (2003). CEO Incentives and corporate social performance. *Journal of Business Ethics, 45*(4), 341-359.

McGuire, J., Dow, S., & Ibrahim, B. (2012). All in the family? Social performance and corporate governance in the family firm. *Journal of Business Research, 65*(11), 1643-1650.

McGuire, J., Oehmichen, J., Wolff, M., & Hilgers, R. (2019). Do contracts make them care? The impact of CEO compensation design on corporate social performance. *Journal of Business Ethics, 157*(2), 375-390.

McKendall, M. A., & Wagner, J. A. (1997). Motive, opportunity, choice and corporate illegality. *Organization Science, 8*(6), 624-647.

Mishra, S., & Modi, S. B. (2013). Positive and negative corporate social responsibility, financial leverage, and idiosyncratic risk. *Journal of Business Ethics, 117*(2), 431-448.

Mousavi, M., Zimon, G., Zimon, G., Salehi, M., Salehi, M., & Stępnicka, N. (2022). The effect of corporate governance structure on fraud and money laundering. *Risks*, *10*(9), 176.

Muller, A., & Kraussl, R. (2011). Doing good deeds in times of need: A strategic perspective on corporate disaster donations. *Strategic Management Journal, 32*(9), 911-929.

Nguyen, P., & Nguyen, A. (2015). The effect of corporate social responsibility on firm risk. *Social Responsibility Journal, 11*(2), 324-339.

Oh, H. (2014). *The asymmetric effects of corporate social responsibility across firms and across time: The role of marketing intensity.* (Ph.D.), University of California, Irvine, Ann Arbor. ProQuest Dissertations & Theses Global A&I: The Humanities and Social Sciences Collection database. (3642942)

Oh, H., Bae, J., Currim, I. S., Lim, J., & Zhang, Y. (2016). Marketing spending, firm visibility, and asymmetric stock returns of corporate social responsibility strengths and concerns. *European Journal of Marketing, 50*(5/6), 838-862.

Oh, W.-Y., Cha, J., & Chang, Y. K. (2017). Does ownership structure matter? The effects of insider and institutional ownership on corporate social responsibility. *Journal of Business Ethics, 146*(1), 111-124.

Oh, W.-Y., Chang, Y. K., & Jung, R. (2018). Experience-based human capital or fixed paradigm problem? CEO tenure, contextual influences, and corporate social (ir)responsibility. *Journal of Business Research, 90*, 325-333.

Oh, W.-Y., Hyun, S., & Park, S. (2016). Top management team pay dispersion and corporate social action: Empirical evidence from US banks. In R. Manos and I. Drori (Eds.), *Corporate Responsibility* (pp. 140-161), Springer.

Oikonomou, I., Brooks, C., & Pavelin, S. (2012). The impact of corporate social performance on financial risk and utility: A longitudinal analysis. *Financial Management, 41*(2), 483-515.

Ormiston, M. E., & Wong, E. M. (2013). License to ill: The effects of corporate social responsibility and CEO moral identity on corporate social irresponsibility. *Personnel Psychology*, *66*(4), 861-893.

O'Sullivan, D., Zolotoy, L., & Fan, Q. (2021). CEO early‐life disaster experience and corporate social performance. *Strategic Management Journal*, *42*(11), 2137-2161.

Park, S., Song, S., & Lee, S. (2017). Corporate social responsibility and systematic risk of restaurant firms: The moderating role of geographical diversification. *Tourism Management, 59*, 610-620.

Park, S. (2019). *CSiR Karma: The stock market reaction to multifaceted firms when a negative issue arises*. Paper presented at the Academy of Management Annual Meeting, Boston, Massachusetts.

Pätäri, S., Arminen, H., Tuppura, A., & Jantunen, A. (2014). Competitive and responsible? The relationship between corporate social and financial performance in the energy sector. *Renewable and Sustainable Energy Reviews, 37*, 142-154.

Perez-Batres, L. A., Doh, J. P., Miller, V. V., & Pisani, M. J. (2012). Stakeholder pressures as determinants of CSR strategic choice: Why do firms choose symbolic versus substantive self-regulatory codes of conduct? *Journal of Business Ethics, 110*(2), 157-172.

Post, C., Rahman, N., & Rubow, E. (2011). Green governance: Boards of directors’ composition and environmental corporate social responsibility. *Business & Society, 50*(1), 189-223.

Price, J. M., & Sun, W. (2017). Doing good and doing bad: The impact of corporate social responsibility and irresponsibility on firm performance. *Journal of Business Research, 80*, 82-97.

Prior, D., Surroca, J., & Tribó, J. A. (2008). Are socially responsible managers really ethical? Exploring the relationship between earnings management and corporate social responsibility. *Corporate Governance: An International Review, 16*(3), 160-177.

Qian, C., Lu, L. Y., & Yu, Y. (2019). Financial analyst coverage and corporate social performance: Evidence from natural experiments. *Strategic Management Journal, 40*(13), 2271-2286.

Qian, L. U. (2014). *Essays on corporate philanthropy after corporate social irresponsible incidents.* (PhD Doctoral dissertation), National University of Singapore,

Ragazou, K., Ragazou, K., Passas, I., & Garefalakis, A. (2022). It is time for anti-bribery: Financial institutions set the new strategic “roadmap” to mitigate illicit practices and corruption in the market. *Administrative Sciences*, *12*(4), 166.

Ren, G., Zeng, P., & Song, T. (2022). Corporate fraud as a negative signal: Implications for firms’ innovation performance. *Business Ethics, the Environment & Responsibility*, *31*(3), 790-808.

Ren, L., Zhong, X., & Wan, L. (2022). Missing analyst forecasts and corporate fraud: Evidence from China. *Journal of Business Ethics*, *181*(1), 171-194.

Rothenhoefer, L. M. (2019). The impact of CSR on corporate reputation perceptions of the public—a configurational multi-time, multi-source perspective. *Business Ethics, 28*(2), 141-155.

Roulet, T. J., & Touboul, S. (2015). The intentions with which the road is paved: Attitudes to liberalism as determinants of greenwashing. *Journal of Business Ethics*, *128*(2), 305-320.

Roush, P. B., Mahoney, L. S., & Thorne, L. (2012). The effects of public pressure on CSR behavior in a capital market experiencing excessive moral debt. *Accounting and the Public Interest*, *12*(1), 87-105.

Sakawa, H., & Watanabel, N. (2022). Accounting frauds and main-bank monitoring in Japanese corporations. *Journal of Business Ethics*, *180*(2), 605-621.

Sheikh, S. (2018). Corporate social responsibility, product market competition, and firm value. *Journal of Economics and Business, 98*, 40-55.

Shi, W., Connelly, B. L., & Sanders, W. G. (2016). Buying bad behavior: Tournament incentives and securities class action lawsuits. *Strategic Management Journal, 37*(7), 1354-1378.

Shi, W., Aguilera, R., & Wang, K. (2020). State ownership and securities fraud: A political governance perspective. *Corporate Governance:* *An International Review*, *28*(2), 157-176.

Shu, H., & Wong, S. M. L. (2018). When a sinner does a good deed: The path‐dependence of reputation repair. *Journal of Management Studies, 55*(5), 770- 808.

Slater, D. J., & Dixon-Fowler, H. R. (2009). CEO international assignment experience and corporate social performance. *Journal of Business Ethics, 89*(3), 473-489.

Song, D. (2014). *The antecedents and consequences of corporate social irresponsibility: Evidence from large U.S. corporations, 1991-2009.* (M.Sc.), University of Manitoba (Canada), Ann Arbor. ABI/INFORM Collection; ProQuest Dissertations & Theses Global A&I: The Humanities and Social Sciences Collection database. (MS26278)

Stewart, O. J. (2016). *A market for vice: The foundations of corporate strategic irresponsibility.* (Ph.D.), The University of North Carolina at Charlotte, Ann Arbor. ABI/INFORM Collection; ProQuest Dissertations & Theses Global A&I: The Humanities and Social Sciences Collection database. (10111931)

Strike, V. M., Gao, J., & Bansal, P. (2006). Being good while being bad: Social responsibility and the international diversification of US firms. *Journal of International Business Studies, 37*(6), 850-862.

Su, F., Feng, X., & Tang, S. (2021). Do site visits mitigate corporate fraudulence? Evidence from China. *International Review of Financial Analysis*, *78*,101940.

Sullivan, B. N., Haunschild, P., & Page, K. (2007). Organizations non gratae? The impact of unethical corporate acts on interorganizational networks. *Organization Science*, *18*(1), 55-70.

Sun, W., & Govind, R. (2022). A new understanding of marketing and “doing good”: Marketing’s power in the TMT and corporate social responsibility. *Journal of Business Ethics*, *176*(1), 89-109.

Swaen, V., Demoulin, N., & Pauwels-Delassus, V. (2021). Impact of customers' perceptions regarding corporate social responsibility and irresponsibility in the grocery retailing industry: The role of corporate reputation. *Journal of Business Research*, 131, 709-721.

Tang, Y., Qian, C., Chen, G., & Shen, R. (2015). How CEO hubris affects corporate social (ir)responsibility. *Strategic Management Journal, 36*(9), 1338-1357.

Tang, Z., & Tang, J. (2016). The impact of competitors–firm power divergence on Chinese SMES’ environmental and financial performance. *Journal of Business Ethics, 136*(1), 147-165.

Teng, C.-C., & Yang, J. J. (2021). Media exposure on corporate social irresponsibility and firm performance. *Pacific-Basin Finance Journal*, *68*, 101604.

Testa, F., Miroshnychenko, I., Barontini, R., & Frey, M. (2018). Does it pay to be a greenwasher or a brownwasher? *Business Strategy and the Environment*, *27*(7), 1104-1116.

Van der Laan, G., Van Ees, H., & Van Witteloostuijn, A. (2008). Corporate social and financial performance: An extended stakeholder theory, and empirical test with accounting measures. *Journal of Business Ethics, 79*(3), 299-310.

Walker, K., Zhang, Z., & Ni, N. (2019). The mirror effect: Corporate social responsibility, corporate social irresponsibility and firm performance in coordinated market economies and liberal market economies. *British Journal of Management, 30*(1), 151-168.

Walls, J. L., Berrone, P., & Phan, P. H. (2012). Corporate governance and environmental performance: Is there really a link? *Strategic Management Journal 33*(8), 885- 913.

Wang, Y. (2019). *Three essays on corporate social responsibility (CSR) of entrepreneurial firms.* (Doctor of Philosophy PhD), University of Manitoba, Winnipeg.

Williams, R. J., Fadil, P. A., & Armstrong, R. W. (2005). Top management team tenure and corporate illegal activity: The moderating influence of board size. *Journal of Managerial Issues*, *17*(4), 479-493.

Wu, J. (2014). The antecedents of corporate social and environmental irresponsibility. *Corporate Social Responsibility and Environmental Management*, *21*(5), 286- 300.

Xu, D., Zhou, K. Z., & Du, F. (2019). Deviant versus aspirational risk taking: The effects of performance feedback on bribery expenditure and R&D Intensity. *Academy of Management Journal, 62*(4), 1226-1251.

Xu, Y., Zhang, L., & Chen, H. (2018). Board age and corporate financial fraud: An interactionist view. *Long Range Planning, 51*(6), 815-830.

Yang, J., & Rhee, J. H. (2020). CSR disclosure against boycotts: Evidence from Korea. *Asian Business and Management*, *19*(3), 311–343.

Yim, H. (2015). *The role of corporate social responsibility to gain sustainable competitive advantage: Three essays.* (Doctor of Philosophy Dissertation), Washington State University, Pullman, US.

Youn, H., Hua, N., & Lee, S. (2015). Does size matter? Corporate social responsibility and firm performance in the restaurant industry. *International Journal of Hospitality Management, 51*, 127-134.

Youn, H., Song, S., Lee, S., & Kim, J.-H. (2016). Does the restaurant type matter for investment in corporate social responsibility? *International Journal of Hospitality Management, 58*, 24-33.

Yuan, Y., Lu, L. Y., Tian, G., & Yu, Y. (2020). Business strategy and corporate social responsibility. *Journal of Business Ethics, 162*(2), 359-377.

Yuan, Y., Tian, G., Lu, L. Y., & Yu, Y. (2019). CEO ability and corporate social responsibility. *Journal of Business Ethics*, *157*(2), 391-411.

Zavyalova, A., Pfarrer, M. D., Reger, R. K., & Hubbard, T. D. (2016). Reputation as a benefit and a burden? How stakeholders' organizational identification affects the role of reputation following a negative event. *Academy of Management Journal*, *59*(1), 253-276.

Zeidan, M. J. (2013). Effects of illegal behavior on the financial performance of US banking institutions. *Journal of Business Ethics, 112*(2), 313-324.

Zeng, T. (2019). Relationship between corporate social responsibility and tax avoidance: International evidence. *Social Responsibility Journal, 15*(2), 244-257.

Zhang, L. (2012). Board demographic diversity, independence, and corporate social performance. *Corporate Governance, 12*(5), 686-700.

Zhang, M., Gong, G., Xu, S., & Gong, X. (2018). Corporate fraud and corporate bond costs: Evidence from China. *Emerging Markets Finance and Trade, 54*(5), 1011-1046.

Zhao, X., Yang, D., Li, Z., & Song, L. (2021). Multiple large shareholders and corporate fraud: Evidence from China. *Frontiers of Business Research in China*, *15*(1),1-21.

Zhong, X., Ren, L., & Ren, G. (2022). Founder CEOs, personal incentives, and corporate social irresponsibility. *Business Ethics, the Environment & Responsibility*, *31*(1), 17-32.

Zhou, N., & Wang, H. (2020). Foreign subsidiary CSR as a buffer against parent firm reputation risk. *Journal of International Business Studies*, *51*(8), 1256-1282.

Zyglidopoulos, S. C., Georgiadis, A. P., Carroll, C. E., & Siegel, D. S. (2012). Does media attention drive corporate social responsibility? *Journal of Business Research, 65*(11), 1622-1627.

1. We use Hunter and Schmidt’s (2014) approach, which in turn draws upon the Spearman–Brown prophecy formula:

   where: $r_{xY}$– the composite correlation coefficient for variable X and Y;
    $r_{{xy}_{i}}$ – the correlation coefficient for variable X and each of the component measures of the composite variable Y;

   $y_{i}$. and $y_{j}$ – each represents one of the component measures of the composite variable Y;

   $r_{{y_{j}y}_{i}}$– the correlation coefficient between two component measures of the composite variable Y ($y_{i}$ and $y_{j}$);

   $n$ – the sample size in the study. [↑](#footnote-ref-1)
